# Ability to use oral fluid and fingerstick HIV self-testing (HIVST) among South African MSM

**DOI:** 10.1371/journal.pone.0206849

**Published:** 2018-11-08

**Authors:** Sheri A. Lippman, Hailey J. Gilmore, Tim Lane, Oscar Radebe, Yea-Hung Chen, Nkuli Mlotshwa, Kabelo Maleke, Albert E. Manyuchi, James McIntyre

**Affiliations:** 1 Center for AIDS Prevention Studies, University of California San Francisco, San Francisco, California, United States of America; 2 Anova Health Institute, Johannesburg, South Africa; 3 Center for Public Health Research, San Francisco Department of Public Health, San Francisco, California, United States of America; 4 School of Public Health & Family Medicine, University of Cape Town, Cape Town, South Africa; New York Blood Center, UNITED STATES

## Abstract

**Background:**

HIV self-testing (HIVST) may increase HIV testing uptake, facilitating earlier treatment for key populations like MSM who experience barriers accessing clinic-based HIV testing. HIVST usability among African MSM has not been explored.

**Methods:**

We assessed usability of oral fluid (OF) and fingerstick (FS; blood) HIVST kits during three phases among MSM with differing degrees of HIVST familiarity in Mpumalanga, South Africa. In 2015, 24 HIVST-naïve MSM conducted counselor-observed OF and FS HIVST after brief demonstration. In 2016 and 2017, 45 and 64 MSM with experience using HIVST in a pilot study chose one HIVST to conduct with a counselor-observer present. In addition to written, the latter group had access to video instructions. We assessed frequency of user errors and reported test use ease, changes in error frequency by phase, and covariates associated with correct usage using log-Poisson and Gaussian generalized estimating equations.

**Results:**

Among OF users (n = 57), 15–30% committed errors in each phase; however, observers consistently rated participants as able to test alone. Among FS users (n = 100), observers noted frequent errors, most commonly related to blood collection and delivery. We found suggestive evidence (not reaching statistical significance) that user errors decreased, with 37.5%, to 28.1%, and 18.2% committing errors in phases I, II, and III, respectively (*p*-value:0.08), however observer concerns remained constant. Ease and confidence using HIVST increased with HIV testing experience. Participants using three HIVST were more likely (RR:1.92, 95% CI:1.32, 2.80) to report ease compared to those without prior HIVST experience. Never testers (RR:0.66, 95% CI:0.44–0.99) reported less ease performing HIVST compared to participants testing in the past six months.

**Conclusions:**

MSM were able to perform the OF test. Fingerstick test performance was less consistent; however preference for fingerstick was strong and performance may improve with exposure and instructional resources. Continued efforts to provide accessible instructions are paramount.

## Introduction

In South Africa, with the largest HIV-positive population globally [1], HIV testing falls far below levels necessary to reduce new infections, particularly among men, who test half as frequently as women [2]. Nationally, approximately one-third of men have never tested [3] and only 37.8% of HIV-positive men are aware of their status [4]. Men who have sex with men (MSM) are no exception: as few as one-fourth of HIV-positive MSM in Mpumalanga Province are aware of their sero-status [5], despite prevalence as high as 28% and incidence of 12.5/ per 100 person years [5, 6]. Data from across sub-Saharan Africa has documented structural barriers to accessing testing services for men. In addition to the cost of travel to clinics, others may be working, traveling for work, or have livelihoods that make clinic attendance difficult. [7, 8] Logistical barriers can be further heightened among men by norms of masculinity that envision clinics and health seeking as feminine [7, 9, 10]. Stigma associated with ‘emasculating’ health seeking behavior and HIV infection associated with risk-taking can deter seeking care [11, 12]. For MSM the experienced stigma and discrimination associated with sexual orientation and HIV act as further deterrents to seeking services in public clinics [13, 14].

HIV self-testing (HIVST) offers an alternative to clinic-based testing, with potential to increase testing uptake and frequency, thus facilitating earlier diagnosis and HIV treatment initiation as well as knowledge of status that encourages safer sexual behavior [15–17]. In 2016 the World Health Organization (WHO) published guidelines [18] recommending HIVST to reach high HIV risk populations, including MSM; the Government of South Africa has echoed support of this strategy in the recently published National Guidelines for HIV Self Screening [19]. Acceptability of HIVST among MSM following self-testing experiences is high for both oral fluid (OF) [15, 20, 21] and blood-based fingerstick (FS) tests [22–24]. Furthermore, HIVST kits, essentially equivalent to antibody lateral flow devices used in healthcare settings, have been found to have high sensitivity and specificity when supervised [25, 26]. Sensitivity may be slightly lower (bottom range in one systematic review was 92.9%) in unsupervised settings [25], signaling some potential for under diagnosis (or false negatives) if not used correctly. A second systematic review noted that individuals’ performance of unassisted HIVST is highly comparable to performance by health care workers, indicating that HIVST can be utilized accurately [27]. Less data is available, however, about unassisted test utilization among key populations in sub-Saharan Africa, who have the greatest need for alternative approaches to HIV testing.

Over several phases of research, we aimed to assess the acceptability, feasibility, uptake, and ability of MSM to conduct OF and FS HIVST in a high prevalence area of South Africa. We have reported on the high acceptability and uptake of HIVST and testing preferences elsewhere [24]. Here we report findings on ability to use HIVST, including frequency and characteristics of user errors and reported ease of use in three MSM groups with varying levels of HIVST exposure. We also assess individuals’ ability to recognize their own capacity for correct utilization and explore factors associated with correct use and comfort with self-testing in order to inform targeted distribution and materials development.

## Materials and methods

### Setting and participants

The study took place among MSM in two high HIV prevalence districts, Gert Sibande and Ehlanzeni, South Africa, which were also known to have sizeable LGBT populations, and where community partnerships for research with these populations were already extant. [28]. The majority of participants were recruited from MSM not known to be HIV-positive who had participated in the Mpumalanga Men’s Study (MpMS) [5], cross-sectional integrated bio-behavioral surveys conducted between 2013–2015 using respondent-driven sampling (RDS) [29]. Additional Ehlanzeni participants were recruited via a newly-conducted RDS scheme, designed to mimic MpMS recruitment. All participants were assigned male sex at birth, ages 18 and over, sexually active with another man in the six months prior to recruitment, who reported HIV-negative or unknown HIV status [5, 24].

### Materials

Two self-testing kits were utilized. The OraQuick HIV-1/2 Rapid Antibody Test (OraSure Technologies, Bethlehem, Pennsylvania, US), approved by the United States FDA for over-the-counter sales in 2012, [30] uses oral fluid swabs from upper and lower gums that is placed into a pre-filled tube of reagent for 20 minutes. Test sensitivity and specificity are 99.3% and 99.8% in a laboratory setting, and 93.0% and 99.98% in self-testing studies [25, 30, 31]. The AtomoRapid HIV-1/2 Antibody Test (Atomo Diagnostics, Sydney, Australia) uses whole blood and has a built-in lancet and specimen collection window. Users prick themselves using the trigger lancet, collect blood (~5 μL) into the onboard collection tube that uses a rotating arm to pull the sample onto a test window, then deliver two drops of reagent, provided in the test package, and wait 15 minutes to read the results. Sensitivity and specificity of the AtomoRapid with professional use are 100.0% and 99.6%, respectively [32]. The AtomoRapid is CE Marked (approved in Europe), has been given ERP Category 3 approval by the Global Fund, and has been submitted for WHO prequalification with approval anticipated later this year; in sum, although not available on shelves, its approval process toward commercial marketing is progressing rapidly. The OraQuick is fully WHO pre-qualified. Both tests are read using a testing window with a control line indicating adequate specimen collection and a test line; user instructions have images of how to interpret the test window for negative, positive, and invalid results.

### Procedures

**Fig 1:**
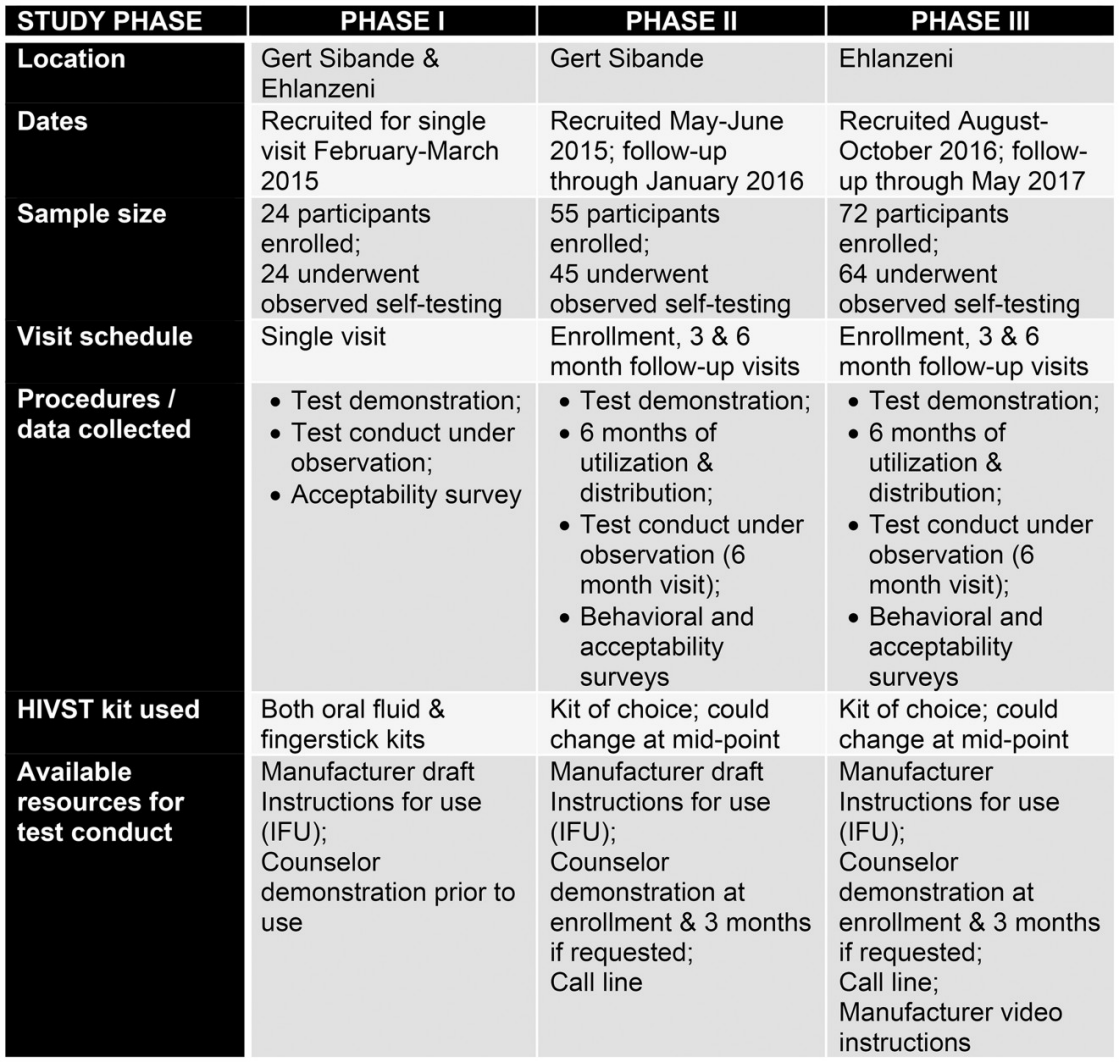
Description of study phases.

This study was conducted among MSM with varying exposure to HIVST over three phases of research (Fig 1).

Phase I was conducted between February and March 2015 and included 24 participants (12 in each site) selected from a random stratified sample of MpMS participants by age (18–24, 25 or older) and education level (some tertiary vs. some secondary). The sample size was selected to ensure that we could identify difficulties with test conduct with diverse users in order to improve instructional materials for the next phases of the study. In a single visit, participants provided written informed consent, viewed a brief demonstration on use of both HIVST kits by a trained counselor, completed an observed testing experience using both OF and FS self-tests, and answered an interviewer-administered acceptability survey. Test-use order was randomly assigned. Counselors recorded testing errors on an observation checklist. Feedback from these sessions informed minor revisions to testing and educational materials for phases II and III of the study, in which we aimed to follow a combined 125 participants between the sites.

Phase II—included recruitment between May and June 2015 of randomly-selected MpMS participants not known to be HIV-positive in the Gert Sibande district. Study staff phoned those selected to assess initial eligibility as well as interest in participating. In total 55 MSM were enrolled, provided written informed consent, and underwent HIV rapid-testing with the counselor to confirm HIV-negative status at enrollment. Participants responded to a brief interviewer-administered behavioral survey, watched a demonstration on how to use both self-tests, and chose which test they would like to take home with them. Each participant received five tests (either OF or FS) to use themselves and to share with partners and others with whom they felt safe and comfortable testing. Participants were provided logs to document test use, a list of local psycho-social and medical resources and referrals, including a 24-hour study hotline, and condoms and lubricant. Participants returned three months following enrollment to deliver test logs, receive up to four additional tests, and complete an acceptability survey. Six months following enrollment, participants returned to deliver test logs, complete a behavioral and acceptability survey that included reported testing experiences, and conduct an HIVST with the kit of their choice under counselor observation. Counselors recorded testing errors on an observation check-list.

Phase III–included recruitment of MpMS participants not known to be HIV-positive as well as recruitment of MSM through the new RDS scheme between August and October 2016 in the district of Ehlanzeni. Participants were screened by phone (MpMS) or in-person (new RDS) to assess eligibility and study interest. In total 72 HIV-negative MSM were consented and enrolled. All procedures were identical to phase II, except that phase III participants were shown manufacturers’ instructional videos about the HIVST at enrollment. Additionally, in phase III the FS manufacturer revised their Instructions for Use (IFU) and participants were provided a weblink to access the instructional videos. Return visits three and six months after enrollment were equivalent to phase II, with the exception that phase III participants could choose to watch the videos at the study office during the three month visit.

Self-test performance was validated in all phases using external quality assurance agents; both the OF and FS tests validated at all time-points on the quality assurance schedule. All procedures were approved by the University of California San Francisco Committee on Human Research, the University of the Witwatersrand’s Human Research Ethics Committee, the Mpumalanga Department of Health and Social Development Research Committee, and the Centers for Disease Control’s Center for Global Health, Human Research Protection Office.

### Measures & analysis

Frequency and type of testing errors observed are described for each test as well as summary indicators regarding a) proportion of participants making an error, b) proportion of participants for whom the observer was concerned about their ability to perform the test alone, and c) proportion of participants who had to repeat the test due to errors rendering the test invalid. Errors are divided into those that are unlikely to, or might, impact test results.

We explored reported self-testing experiences using Likert-scale questions on the acceptability (phase I) and three- and six-month surveys (phases II-III) regarding the ease or difficulty of collecting the sample, conducting test procedures, and interpreting test results, referent to the test utilized. We also asked about participants’ confidence in completing the test correctly and trust of results. We created a summary indicator variable for ‘ease of and comfort with testing’ that included those who responded ‘somewhat easy’ or ‘very easy’ and ‘somewhat comfortable’ or ‘very comfortable’ to all the above questions. During the observed test in phases II-III, participants were given an option to use either test kit; as a result some participants utilized a kit during observation that they had not used previously. We restricted data on reported experience to include only participants who were observed and reported on the same test kit (e.g. we assess observed OF with reported experience using OF) to facilitate comparing correct use during observation and reported ease and comfort, resulting in fewer observations in the reported experience data.

Observation check-list data was captured on paper, entered into Excel, and content re-verified. Surveys were conducted in QDS (Questionnaire Development System) and exported to R (R Foundation for Statistical Computing, Vienna, Austria) for analysis. Frequency tables were generated to describe population demographics and behaviors, observed testing errors, and testing experiences by phase. We examined associations between user characteristics and research phase using Fisher’s exact tests (Table 1). We used Gaussian generalized estimating equations (GEE) to test for trends in testing errors and observer concerns over the phases, treating phase as a linear exposure (Table 2). We also assessed characteristics associated with correct usage during observation and reported ease and comfort testing. For these analysis with binary outcomes, we used a log-Poisson GEE when data included repeated measures (including phase I participants), and used a Poisson-based generalized linear model with a log link function when repeated measures were not involved (no phase I observations) (Table 3). Finally, we assessed participants’ observed ability to conduct the test as compared to their stated ease and comfort in order to explore whether participants can recognize their own ability to conduct self-tests.

**Table 1:** Baseline characteristics of HIVST study populations participating in observed testing, Mpumalanga, South Africa.

Respondent characteristics	Phase 1: 2015 HIVST naïve (n = 24)		Phase 2: 2016 HIVST experienced (Gert Sibande) (n = 45)		Phase 3: 2017 HIVST experienced (Ehlanzeni) (n = 64)	
	n	%	n	%	n	%
Age						
18–24	10	41.7	30	66.7	44	68.8
25–39	14	58.3	15	33.3	20	31.2
Highest level of education*						
Primary or secondary	9	37.5	16	35.6	40	62.5
Matric (high school graduate)	10	41.7	23	51.1	18	28.1
Some college or technical school	5	20.8	6	13.3	6	9.4
Paid work in the past six months?						
Yes	11	45.8	13	28.9	22	34.4
No	13	54.2	32	71.1	42	65.6
Sexual identity*						
Gay/homosexual	12	50.0	7	15.6	26	40.6
Bisexual	10	41.7	34	75.6	38	59.4
Straight	2	8.3	3	6.7	0	0
Transgender ^¥^	0	0	1	2.2	0	0
HIV testing history ^†^*						
Never tested	7	29.2	7	15.6	9	14.1
Tested in past 6 months	7	29.2	9	20.0	36	56.2
Tested 6–12 months ago	6	25.0	24	53.3	5	7.8
Tested >12 months ago	4	16.7	5	11.1	14	21.9
Current regular male sexual partner						
Yes	17	70.8	35	77.8	54	84.4
No	7	29.2	10	22.2	10	15.6
Number of partners in the last six months						
0	0	0	2	4.4	1	1.6
1	12	50	30	66.7	37	57.8
≥2	12	50	13	28.9	26	40.6

* differences by study phase (p ≤ .05);

^¥^ Participant identified their sexual identity as transgender;

^†^ Reported HIV testing behaviors prior to participation in our HIV testing research initiatives, including MpMS.

**Table 2:** Participant performance utilizing HIV self-tests under observed and non-observed conditions and reported acceptability indicators among MSM in Mpumalanga, South Africa.

	Phase 1^‡^: Self-test naïve; following brief instructions				Phase 2^§^: 6 months following HIVST distribution				Phase 3^¶^: 6 months following HIVST distribution			
**Observed self-testing**	**Oral** **(n = 24)**		**Blood**^#^ **(n = 24)**		**Oral** **(n = 13)**		**Blood** **(n = 32)**		**Oral** **(n = 20)**		**Blood** **(n = 44)**	
	n	%	n	%	n	%	n	%	n	%	n	%
Minor observed errors / challenges												
Did procedures out of order	1	4.2	2	8.3	1	7.7	0	0	4	20.0	0	0
Didn’t place tube in stand properly	4	16.7			1	7.7			2	10.0		
Forgot to clean finger			1	4.2			1	3.1			0	0
Spilled test tube contents (partial)	1	4.2			0	0			1	5.0		
Major observed errors / challenges												
Touched pad	0	0			1	7.7			0	0		
Errors timing results (waits too little / too long)	1	4.2	0	0	0	0	1	3.1	0	0	0	0
Lancet error (failed to remove tab / ejected lancet)			4	16.7			4	12.5			5	11.4
Error delivering blood to kit			5	20.8			4	12.5			3	6.8
Diluent Errors (too few / many drops)			4	16.7			0	0			1	2.3
Participants committing errors	4	16.7	9	37.5	2	15.4	9	28.1	6	30.0	8	18.2
Observer concern with participant’s ability to perform test alone	0	0	4	16.7	0	0	5	15.6	0	0	6	13.6
Test invalid; had to repeat test	0	0	1	4.2	0	0	0	0	0	0	0	0
Tested positive	0	0	1	4.2	0	0	2	6.2	0	0	4	9.1
**Reported self-testing experience**	**N = 24**		**N = 23**^#^		**N = 8** ^¶^		**N = 30** ^¶^		**N = 17** ^¶^		**N = 39**^¶^	
Ease/difficulty collecting sample?												
Very easy	22	91.7	16	69.6	8	100	21	70.0	17	100	34	87.2
Somewhat easy	1	4.2	3	13.0	0	0	9	30.0	0	0	4	10.3
Difficult (somewhat + very)	1	4.2	4	17.4	0	0	0	0	0	0	1	2.6
Ease/difficulty conducting test?												
Very easy	20	83.3	19	82.6	7	87.5	20	66.7	16	94.1	34	87.2
Somewhat easy	2	8.3	3	13.0	1	12.5	10	33.3	1	5.9	5	12.8
Difficult (somewhat + very)	2	8.3	1	4.3	0	0	0	0	0	0	0	0
Reported ease/difficulty interpreting result?												
Very easy	19	79.2	20	87.0	8	100	29	96.7	17	100	38	97.4
Somewhat easy	4	16.7	2	8.7	0	0	1	3.3	0	0	0	0
Difficult (somewhat + very)	1	4.2	1	4.3	0	0	0	0	0	0	1	2.6
Trust results?												
Yes	24	100	23	100	7	87.5	30	100	17	100	39	100
No	0	0	0	0	1	12.5	0	0	0	0	0	0
Confident you did test correctly?												
Very confident	21	87.5	20	87.0	8	100	28	93.3	17	100	37	94.9
Somewhat confident	3	12.5	3	13.0	0	0	2	6.7	0	0	2	5.1
Not confident	0	0	0	0	0	0	0	0	0	0	0	0

^‡^ Participants used both kits;

^§^ Participants used kit of their choosing;

^¶^ Includes participants reporting experiences on test kit used during observation;

^#^ One participant in phase I was not asked about blood testing experiences due to interviewer error.

**Table 3:** Factors associated with correct HIVST usage and reported comfort and ease among MSM in Mpumalanga, South Africa.

Respondent characteristics	Correct usage during observed test N = 157				Reported comfort + ease N = 141^			
**All phases**	n	%	RR (95% CI)	p-value	n	%	RR (95% CI)	p-value
Age								
18–24	94	87.2	1.00	0.09	83	63.9	1.00	0.97
25–39	63	76.2	0.87 (0.74,1.02)		58	65.5	1.00 (0.77, 1.28)	
Highest level of education								
Primary or secondary	74	82.4	1.00	0.97	62	74.2	1.00	0.20
Matric (high school graduate)	61	83.6	1.01 (0.87, 1.18)		57	59.6	0.83 (0.63, 1.08)	
Some college or technical school	22	81.8	0.99 (0.80, 1.23)		22	50.0	0.74 (0.48, 1.13)	
Recruitment Site								
Gert Sibande	69	78.3	1.00	0.19	61	55.7	1.00	0.05
Ehlanzeni	88	86.4	1.11 (0.95, 1.28)		80	71.2	1.30 (1.0, 1.71)	
Paid work in the past six months?								
No	100	85.0	1.00	0.36	93	66.7	1.00	0.34
Yes	57	78.9	0.93 (0.79,1.09)		48	60.4	0.87 (0.65, 1.16)	
Sexual identity								
Gay/homosexual	57	84.2	1.00	0.65	54	59.3	1.00	0.26
Bisexual	92	83.7	0.99 (0.87,1.14)		81	70.4	1.16 (0.89, 1.51)	
Straight	7	57.1	0.68 (0.30, 1.53)		5	20.0	0.37 (0.06, 2.17)	
Transgender^¥^	1	100	-		1	100	-	
HIV testing history								
Tested in past 6 months	59	84.7	1.00	0.60	53	77.4	1.00	0.07
Tested 6–12 months ago	41	87.8	1.04 (0.89, 1.21)		38	63.2	0.82 (0.61, 1.08)	
Tested >12 months ago	27	77.8	0.92 (0.73, 1.15)		23	52.2	0.68 (0.45, 1.04)	
Never tested	30	76.7	0.90 (0.71, 1.15)		27	51.9	0.66 (0.44, 0.99)	
Number of self-testing kits used								
0	52	78.8	1.00	0.68	47	48.9	1.00	<0.01
1–2	91	84.6	1.07 (0.91, 1.27)		80	68.8	1.42 (0.97, 2.07)	
3	14	85.7	1.09 (0.84, 1.40)		14	92.9	1.92 (1.32, 2.80)	
	**N = 109**				**N = 94**			
**Phases II & III only**^$^	n	%	RR (95% CI)	p-value	n	%	RR (95% CI)	p-value
Number of HIV tests ever								
0	5	100	1.00	1.0	5	100	1.00	0.24
1–5	57	84.2	0.84 (0.34, 2.12)		46	76.1	0.76 (0.30, 1.94)	
6+	47	83.0	0.83 (0.33, 2.11)		43	65.1	0.65 (0.25, 1.69)	
Use of cell phone to access internet								
Yes	79	86.1	1.00	0.55	69	73.9	1.00	0.61
No	30	80.0	0.93 (0.58,1.48)		25	68.0	0.92 (0.53, 1.59)	
Used HIVST with someone else there?								
Yes	26	84.6	1.00	1.00	23	78.3	1.00	0.60
No	83	84.3	1.00 (0.62, 1.61)		71	70.4	0.90 (0.53, 1.54)	

*^* Reported comfort/ease limited to those reporting on the same test used during observation, resulting in fewer data points: some participants chose to utilize tests for observation that they had not reported using during the study;

^¥^ Participant identified their sexual identity as transgender;

^$^ questions not asked during phase I.

## Results

All men reached for recruitment during phase I agreed to participate in the study, resulting in 24 enrolled with complete study procedures. Among 58 eligible participants contacted in phase II, 55 (95%) enrolled; follow-up data (at three months, six months, or both) was captured for 51 participants, with 45 completing observed testing. In phase III, 72 (90%) of 80 eligible potential participants enrolled, with follow-up data on 65 participants and observed testing experiences for 64 (Fig 1).

While there was no upper age bound for recruitment, the RDS samples were all under the age of 40. Most participants did not have paid work and were under the age of 25, with 41.7%, 66.7%, and 68.8% of the phase I-III samples, respectively, being between the ages of 18–24 (Table 1). Most participants identified as bisexual or gay, though the distribution of sexual identities varied across phases. Testing history also varied by phase. Prior to participation in the joint research initiatives (MpMS and the HIVST study), the majority of participants had HIV-tested before, largely within the last 12 months, though never testing was as high as 29% in phase I, and only 14% in phase III. Between 71% and 84% of participants reported having a regular male sexual partner at the time of study enrollment. There were no differences in age, education, sexual identity, or HIV testing history between those returning for follow-up and those who did not (data not shown).

### Test usability

Observed OF testing included 57 tests, with 24, 13, and 20 participants observed in phases I-III, respectively. Most recorded errors were minor (Table 2), the most common being conducting procedures out of order (e.g. collecting the sample before opening the tube) and not placing the tube securely. All users of the OF tests had valid results. Observers were universally confident that users would be able to conduct the OF test correctly in an unobserved setting. Frequency of errors did not decrease over the study phases. Similarly, among the 24 (phase I), eight (phase II), and 18 (phase III) participants observed conducting an OF and who reported on their OF self-testing experience, all but one in phase I reported it was very easy to collect the sample and interpret the result, and only two in phase I reported the test being difficult to conduct. One participant in phase II stated they did not trust the result. Participants were confident in their ability to use the OF test: 87.5% in phase I and 100% in phases II-III felt very confident they used the OraQuick test correctly.

Observed FS testing included 100 user-conducted tests, including 24, 32, and 44 participants in phases I-III, respectively. Major errors with the FS test were more frequent as compared to OF. The most common included lancet errors (n = 13), errors delivering blood to the kit (n = 12), and diluent errors (n = 5). During phase I, 16 errors were made by nine individuals (37.5%) and counselors noted concerns about four participants (16.7%) being able to perform the test alone. In phases II-III, participants committed fewer errors on the FS test, with nine (28.1%) committing errors in phase II and eight (18.2%) in phase III (Table 2). While fewer people committed errors in the later phases of the study (test for trend *p*-value: 0.08), the proportion of participants for whom observers registered concerns remained consistently between 13% and 17% over all phases. While 13 participants had to use an external lancet, only one participant (phase I) had to repeat the entire test due to invalid results; this occurred after delivering only one drop of diluent (vs. recommended two). Overall seven participants tested positive on the blood test and were referred to a local MSM-friendly clinic [33]. Among 92 participants who were observed conducting the FS test and reported FS testing experiences, four (17.4%) during phase I and one in phase III (2.6%) reported difficulty collecting the sample and interpreting the results. Only one participant in phase I also reported difficulty conducting the test. All participants trusted the blood test results and 87%, 93%, and 95% felt very confident they used the blood test correctly in phases I-III, respectively (Table 2).

Among participants who were observed utilizing the OF and reported on using OF tests, level of agreement between reported ease and comfort and correct observed use was high. Only one participant who reported ease and comfort was observed making a mistake (positive predictive value (PPV) of reported ability 97.1%). Participants’ ability to discern their own skill on the FS test was lower, with ten participants who reported ease and comfort having an observed mistake by the counselor (PPV 82.1%).

Younger age was the only characteristic marginally associated with correct observed use (p = .09); education level was not associated with correct use (Table 3). Participant characteristics associated with stated ease and comfort included Ehlanzeni recruitment site (p = .05) and having more testing experiences. Compared to those who had tested in the past six months, those who had never HIV-tested were 66% less likely (RR 0.66, 95% 0.44–0.99) to report ease and comfort with test use. Similarly, compared to those who had tested in the past six months, those who had tested more than one year ago reported less ease and comfort (RR 0.68, 95% CI 0.45–1.04), though this did not reach statistical significance. Additionally, those who had conducted three or more self-tests were more likely to report ease and comfort self-testing as compared to those who had not used HIVST prior to observation (RR: 1.92, 95% CI 1.32–2.80).

## Discussion

We found that overall South African MSM with differing degrees of experience and familiarity with HIVST were universally able to successfully use OraQuick OF test. Most errors were unlikely to render tests invalid. Participants were confident about using the OF tests, finding them easy to use. Our findings are comparable to recent studies conducted in sub-Saharan Africa. In a general population in Kwa-Zulu Natal, only .09% of OF testers had to repeat testing due to errors following a self-testing demonstration [34]. In a Ugandan fishing community [35], observers noted errors among 19% of participants but most tests were still successfully conducted, similar to our findings. However in the Uganda study, only three-quarters of users found the test easy [35], notably lower than our sample; however our participants appeared to test more frequently and therefore may have more comfort testing. Fewer user errors were reported in general populations studies in Kenya [36] and Malawi [37], with similar levels of acceptability and reported ease as our findings. Additionally, studies conducted in Africa have demonstrated a sensitivity of self-conducted OF testing at 90% or higher with specificity consistently at or above 95% [34–38], with one study conducted among healthcare workers in South Africa demonstrating lower sensitivity, primarily due to poor reading of (and lack of instructions around) weak positives [39].

While most MSM participants in our study could also perform the Atomo Diagnostics FS test, errors were more common, resulting in about one-sixth of users rated as unable to conduct the test alone. Though there has been collectively less research in Africa regarding blood-based HIVST, a recent study undertaken in the Central African Republic using a different FS test [40] found that while almost all participants could conduct the test, only 78.2% of lay users performed a FS test without error. Also similar to our findings, participants had the most trouble using the lancet. [40] A study in Cape Town [41] assessed usability of the Atomo test among young people (16–24 years), noting errors in only 3.6% of users, a much lower prevalence of errors as compared to our findings. This may be due to more intensive training prior to use; our training was a brief demonstration of the test and a review of the IFU. Like our sample, errors in Cape Town were largely related to blood draw and delivery to the device. In a multi-country study using 4 fingerstick HIVST prototypes, Peck et al. found that less than half performed the tests without errors, with only 61% able to collect a sample [42]; however users were not given training or instructions prior to use and the written instructions provided were not final manufacturer instructions.

Overall, our study and the majority of the evidence gathered around HIVST usability in Africa indicate that FS tests will require more instruction than what is currently included in the package insert. We did find evidence that fewer participants made errors on FS testing and more reported ease and comfort with FS testing in the later cohorts with additional degrees of exposure to HIVST and access to instructional videos. This was also observed in the Kenya OF testing study; the authors noted that confidence in the ability to perform and interpret tests appeared to increase with testing experience [36]. These findings highlight the importance of continuing to develop resources for blood-based HIVST to ensure correct use, particularly in light of the evidence of improved test performance when using blood-based specimens [43, 44]. We believe that the instructional videos helped users in phase III, but note that our population did not report accessing the videos outside of the study office, as few had internet access or mobile device data plans. As a result, resources are needed that do not require internet connections or use large amounts of mobile data. Additionally, while we provided a hotline, calls for assistance were limited to two participants who called for further instruction, suggesting that while important to offer, participants may not utilize such a resource.

In terms of associations with correct test use and comfort conducting the tests, we found some evidence that younger participants conducted the tests with fewer errors, which has been noted in previous research [45]. We also found that recent exposure to clinic-based testing and more exposure to self-testing resulted in more reported confidence in conducting HIVST, though the same was not true with observed test performance. This increased confidence could be due to having a recent seronegative test result and therefore feeling less nervous and more focused while conducting HIVST. Increased comfort with increased HIVST exposure is also likely a result of gaining practice and familiarity with procedures, including steps such as pricking the finger, which can be daunting. Generally HIVST testing confidence has been demonstrated to be higher than the proportion of people conducting the tests correctly [26]. Our findings and past research [42] suggests that users overestimate their abilities to conduct HIVST alone, making improved instructional resources even more urgent.

This is among the first studies of both OF and FS HIVST use among sub-Saharan African MSM. Limitations include potential biases inherent in the use of counselor-observers, as being observed could lead to participant discomfort. Additionally, the sample size is small and skewed towards a young MSM population; however, results revealed a number of important findings and next steps towards improving diagnostic capacity in this high risk population.

## Conclusions

Against the backdrop of both structural barriers to clinic-based testing as well as feared and experienced stigmatization in public health care settings, [13, 14] MSM have evidenced a clear need and reported a resounding support for HIVST in two high prevalence districts of South Africa. While utilization errors were observed, particularly on the fingerstick test, increasing experience and exposure to HIVST is likely to decrease utilization errors. Further optimization of test devices to facilitate sample collection, improved supporting materials and instructions for use, and use of demonstration videos and Smartphone applications that can be downloaded and used offline (i.e. do not require ongoing use of network data), should improve usability and decrease frequency of errors. Rapid progress in HIVST quality will also be spurred by the growing number products entering the market and the increasing number of countries, including South Africa, producing regulatory policies [46] and stepping up monitoring systems to ensure device quality and appropriate instructions for use. Even with areas for improvement, HIVST are safe, acceptable, and feasible to use and will benefit populations like MSM that require alternatives to clinic-based HIV testing.

## References

[1] UNAIDS. Global Report: UNAIDS Report on the Global AIDS Epidemic. Geneva: UNAIDS, 2010.

[2] South African National AIDS Council. HCT and ART expansion Results. Pretoria: Aug, 2011.

[3] South Africa Demographic and Health Survey 2016: Key Indicators Report. Pretoria, South Africa: National Department of Health; Statistics South Africa, 2017.

[4] Shisana O, Rehle T, Simbayi LC, Zuma K, Jooste S, Zungu N, et al. South African National HIV Prevalence, Incidence and Behaviour Survey, 2012. Cape Town: HSRC Press, 2014. 10.2989/16085906.2016.1153491 27002359

[5] Lane T, Osmand T, Marr A, Shade SB, Dunkle K, Sandfort T, et al The Mpumalanga Men’s Study (MPMS): results of a baseline biological and behavioral HIV surveillance survey in two MSM communities in South Africa. PLoS One. 2014;9(11):e111063 10.1371/journal.pone.0111063 . 25401785 PMC4234301

[6] Lane T, Osmand T, Marr A, Struthers H, McIntyre JA, Shade SB. Brief Report: High HIV Incidence in a South African Community of Men Who Have Sex With Men: Results From the Mpumalanga Men’s Study, 2012–2015. Journal of acquired immune deficiency syndromes. 2016;73(5):609–11. 10.1097/QAI.0000000000001162 . 27851715

[7] Camlin CS, Ssemmondo E, Chamie G, El Ayadi AM, Kwarisiima D, Sang N, et al Men "missing" from population-based HIV testing: insights from qualitative research. AIDS care. 2016;28 Suppl 3:67–73. 10.1080/09540121.2016.1164806 . 27421053 PMC5749410

[8] Sharma M, Barnabas RV, Celum C. Community-based strategies to strengthen men’s engagement in the HIV care cascade in sub-Saharan Africa. PLoS Med. 2017;14(4):e1002262 10.1371/journal.pmed.1002262 . 28399122 PMC5388461

[9] Skovdal M, Campbell C, Madanhire C, Mupambireyi Z, Nyamukapa C, Gregson S. Masculinity as a barrier to men’s use of HIV services in Zimbabwe. Globalization and health. 2011;7:13 10.1186/1744-8603-7-13 . 21575149 PMC3107786

[10] DiCarlo AL, Mantell JE, Remien RH, Zerbe A, Morris D, Pitt B, et al ’Men usually say that HIV testing is for women’: gender dynamics and perceptions of HIV testing in Lesotho. Culture, health & sexuality. 2014;16(8):867–82. 10.1080/13691058.2014.913812 . 24854495 PMC4116609

[11] Mooney AC, Gottert A, Khoza N, Rebombo D, Hove J, Suarez AJ, et al Men’s Perceptions of Treatment as Prevention in South Africa: Implications for Engagement in HIV Care and Treatment. AIDS education and prevention: official publication of the International Society for AIDS Education. 2017;29(3):274–87. 10.1521/aeap.2017.29.3.274 . 28650225 PMC6686680

[12] Treves-Kagan S, Steward WT, Ntswane L, Haller R, Gilvydis JM, Gulati H, et al Why increasing availability of ART is not enough: a rapid, community-based study on how HIV-related stigma impacts engagement to care in rural South Africa. BMC public health. 2016;16:87 10.1186/s12889-016-2753-2 . 26823077 PMC4730651

[13] Lane T, Mogale T, Struthers H, McIntyre J, Kegeles SM. "They see you as a different thing": the experiences of men who have sex with men with healthcare workers in South African township communities. Sex Transm Infect. 2008;84(6):430–3. 10.1136/sti.2008.031567 . 19028941 PMC2780345

[14] Rispel LC, Metcalf CA, Cloete A, Moorman J, Reddy V. You become afraid to tell them that you are gay: health service utilization by men who have sex with men in South African cities. J Public Health Policy. 2011;32 Suppl 1:S137–51. Epub 2011/07/07. 10.1057/jphp.2011.29 . 21730987

[15] Carballo-Dieguez A, Frasca T, Balan I, Ibitoye M, Dolezal C. Use of a Rapid HIV Home Test Prevents HIV Exposure in a High Risk Sample of Men Who Have Sex With Men. AIDS Behav. 2012;16(7):1753–60. Epub 2012/08/16. 10.1007/s10461-012-0274-2 . 22893194 PMC3458207

[16] Frasca T, Balan I, Ibitoye M, Valladares J, Dolezal C, Carballo-Dieguez A. Attitude and behavior changes among gay and bisexual men after use of rapid home HIV tests to screen sexual partners. AIDS Behav. 2014;18(5):950–7. 10.1007/s10461-013-0630-x . 24077975 PMC3969408

[17] Johnson C, Baggaley R, Forsythe S, van Rooyen H, Ford N, Napierala Mavedzenge S, et al Realizing the potential for HIV self-testing. AIDS Behav. 2014;18 Suppl 4:S391–5. 10.1007/s10461-014-0832-x . 24986599

[18] World Health Organization. Guidelines on HIV Testing Services and Partner Notification: Supplement to Consolidated Guidelines on HIV Testing Services Geneva, Switzerland: World Health Organization, 2016 12. 27977094

[19] Department of Health Republic of South Africa. National HIV Self Screening Guidelines. Pretoria: National Department of Health, 2018.

[20] Woods WJ, Lippman SA, Agnew E, Carroll S, Binson D. Bathhouse distribution of HIV self-testing kits reaches diverse, high-risk population. AIDS care. 2016;28 Suppl 1:111–3. 10.1080/09540121.2016.1146399 . 26883730 PMC4828605

[21] Katz D, G M, Hughes J, Farquhar C, Stekler J. HIV self-testing increases HIV testing frequency among high-risk men who have sex with men: a randomized controlled trial. International AIDS Society Vancouver, Canada, 2015. 10.1097/QAI.0000000000001709 PMC6037557 29697595

[22] Volk JE, Lippman SA, Grinsztejn B, Lama JR, Fernandes NM, Gonzales P, et al Acceptability and feasibility of HIV self-testing among men who have sex with men in Peru and Brazil. International journal of STD & AIDS. 2015 10.1177/0956462415586676 . 25971262 PMC4643427

[23] Zhong F, Tang W, Cheng W, Lin P, Wu Q, Cai Y, et al Acceptability and feasibility of a social entrepreneurship testing model to promote HIV self-testing and linkage to care among men who have sex with men. HIV medicine. 2017;18(5):376–82. 10.1111/hiv.12437 . 27601301 PMC5340630

[24] Lippman SA, Lane T, Radebe O, Gilmore HJ, Chen Y-H, Mlotshwa N, et al High Acceptability and Increased HIV Testing Frequency Following Introduction of HIV Self-Testing and Network Distribution among South African MSM JAIDS. 2018;77(3):279–87. 29210826 10.1097/QAI.0000000000001601 PMC5807184

[25] Pant Pai N, Sharma J, Shivkumar S, Pillay S, Vadnais C, Joseph L, et al Supervised and unsupervised self-testing for HIV in high- and low-risk populations: a systematic review. PLoS medicine. 2013;10(4):e1001414 Epub 2013/04/09. 10.1371/journal.pmed.1001414 . 23565066 PMC3614510

[26] Stevens DR, Vrana CJ, Dlin RE, Korte JE. A Global Review of HIV Self-testing: Themes and Implications. AIDS Behav. 2017; [Epub ahead of print]. 10.1007/s10461-017-1707-8 . 28155039 PMC5910655

[27] Figueroa C, Johnson C, Ford N, Sands A, Dalal S, Meurant R, et al Reliability of HIV rapid diagnostic tests for self-testing compared with testing by health-care workers: a systematic review and meta-analysis. The lancet HIV. 2018 10.1016/S2352-3018(18)30044-4 . 29703707 PMC5986793

[28] National Department of Health. The National Antenatal Sentinel HIV and Syphilis Prevalence Survey, South Africa, 2011. Pretoria: 2012.

[29] Heckathorn D. Respondent-driven sampling II: Deriving valid population estimates from chain-referral samples of hidden populations. SocProbl. 2002;49(11–34).

[30] US Food and Drug Administration (USFDA). OraQuick In-Home HIV Test. July 3, 2012. on line at: http://www.fda.gov/BiologicsBloodVaccines/BloodBloodProducts/ApprovedProducts/PremarketApprovalsPMAs/ucm310436.htm.

[31] Estem KS, Catania J, Klausner JD. HIV Self-Testing: a Review of Current Implementation and Fidelity. Current HIV/AIDS reports. 2016;13(2):107–15. 10.1007/s11904-016-0307-y . 26879653

[32] Atomo Diagnostics Pty Ltd. Technical Information Pack—AtomoRapid HIV. Evaluated by the National Institute for Communicable Diseases in South Africa: 2014 Jan; 3rd Generation; Kit Lot Number 2013111809.

[33] Anova Health Institute. Health 4 Men: http://www.health4men.co.za/ 2015.

[34] Martinez Perez G, Steele SJ, Govender I, Arellano G, Mkwamba A, Hadebe M, et al Supervised oral HIV self-testing is accurate in rural KwaZulu-Natal, South Africa. Trop Med Int Health. 2016;21(6):759–67. 10.1111/tmi.12703 . 27098272

[35] Asiimwe S, Oloya J, Song X, Whalen CC. Accuracy of Un-supervised Versus Provider-Supervised Self-administered HIV testing in Uganda: A Randomized Implementation Trial. AIDS Behav. 2014;(18):2477–84. Epub 2 April 2014. 10.1007/s10461-014-0765-4 24691923 PMC4183743

[36] Kurth AE, Cleland CM, Chhun N, Sidle JE, Were E, Naanyu V, et al Accuracy and Acceptability of Oral Fluid HIV Self-Testing in a General Adult Population in Kenya. AIDS Behav. 2016;20(4):870–9. 10.1007/s10461-015-1213-9 . 26438487 PMC4799243

[37] Choko AT, Desmond N, Webb EL, Chavula K, Napierala-Mavedzenge S, Gaydos CA, et al The uptake and accuracy of oral kits for HIV self-testing in high HIV prevalence setting: a cross-sectional feasibility study in Blantyre, Malawi. PLoS Med. 2011;8(10):e1001102 10.1371/journal.pmed.1001102 . 21990966 PMC3186813

[38] Choko AT, Taegtmeyer M, MacPherson P, Cocker D, Khundi M, Thindwa D, et al Initial Accuracy of HIV Rapid Test Kits Stored in Suboptimal Conditions and Validity of Delayed Reading of Oral Fluid Tests. PLoS One. 2016;11(6):e0158107 10.1371/journal.pone.0158107 . 27336161 PMC4918937

[39] Pant Pai N, Behlim T, Abrahams L, Vadnais C, Shivkumar S, Pillay S, et al Will an unsupervised self-testing strategy for HIV work in health care workers of South Africa? A cross sectional pilot feasibility study. PLoS One. 2013;8(11):e79772 10.1371/journal.pone.0079772 . 24312185 PMC3842310

[40] Gresenguet G, Longo JD, Tonen-Wolyec S, Mboumba Bouassa RS, Belec L. Acceptability and Usability Evaluation of Finger-Stick Whole Blood HIV Self-Test as An HIV Screening Tool Adapted to The General Public in The Central African Republic. Open AIDS J. 2017;11:101–18. 10.2174/1874613601711010101 . 29290887 PMC5730956

[41] Smith P, Wallace M, Bekker LG. Adolescents’ experience of a rapid HIV self-testing device in youth-friendly clinic settings in Cape Town South Africa: a cross-sectional community based usability study. J Int AIDS Soc. 2017;19(1):1–6. 10.7448/IAS.19.1.21111 . 28406597 PMC5380981

[42] Peck RB, Lim JM, van Rooyen H, Mukoma W, Chepuka L, Bansil P, et al What Should the Ideal HIV Self-Test Look Like? A Usability Study of Test Prototypes in Unsupervised HIV Self-Testing in Kenya, Malawi, and South Africa. AIDS Behav. 2014;18 Epub 20 June 2014. 10.1007/s10461-014-0818-8 24947852

[43] Pant Pai N, Balram B, Shivkumar S, Martinez-Cajas JL, Claessens C, Lambert G, et al Head-to-head comparison of accuracy of a rapid point-of-care HIV test with oral versus whole-blood specimens: a systematic review and meta-analysis. Lancet Infect Dis. 2012;12(5):373–80. 10.1016/S1473-3099(11)70368-1 . 22277215

[44] Stekler JD, O’Neal JD, Lane A, Swanson F, Maenza J, Stevens CE, et al Relative accuracy of serum, whole blood, and oral fluid HIV tests among Seattle men who have sex with men. Journal of clinical virology: the official publication of the Pan American Society for Clinical Virology. 2013;58 Suppl 1:e119–22. 10.1016/j.jcv.2013.09.018 . 24342471 PMC3867744

[45] de la Fuente L, Rosales-Statkus ME, Hoyos J, Pulido J, Santos S, Bravo MJ, et al Are participants in a street-based HIV testing program able to perform their own rapid test and interpret the results? PLoS One. 2012;7(10):e46555 10.1371/journal.pone.0046555 . 23056342 PMC3466298

[46] HIV Self-testing Clearinghouse. HIVST.org: HIV self-testing research and policy hub 2018 [accessed March 9, 2018]. http://www.hivst.org/policy.

